# Measurement invariance of the patient health questionnaire-9 depression scale in a nationally representative population-based sample

**DOI:** 10.3389/fpsyg.2023.1217038

**Published:** 2023-08-31

**Authors:** Eun-Hyun Lee, Eun Hee Kang, Hyun-Jung Kang, Hyun Young Lee

**Affiliations:** ^1^Graduate School of Public Health, Ajou University, Suwon, Republic of Korea; ^2^Clinical Trial Center, Ajou University Hospital, Suwon, Republic of Korea

**Keywords:** depressive symptoms, measurement invariance, network psychometrics, structural validity, internal consistency, questionnaire

## Abstract

The Patient Health Questionnaire-9 (PHQ-9) is widely used to measure the severity of depressive symptoms and to screen for depressive disorder, but its measurement invariance has received little research attention. The aim of this study was to assess the measurement invariance of the PHQ-9 across various sociodemographic and medical-condition groups. The structural validity and internal consistency of the PHQ-9 were also assessed as the prerequisite properties for measurement invariance. This study was conducted using data from the Korea National Health and Nutrition Examination Survey. The included participants comprised 5,347 people older than 19 years. Exploratory graph analysis (EGA) and confirmatory factor analysis (CFA) were performed to determine structural validity, and the omega coefficient ( ω ) was used to determine internal consistency. Measurement invariance (configural, metric, and scalar invariance) was evaluated using multigroup confirmatory factor analysis (MGCFA). The single structural model of the PHQ-9 that was validated by EGA was also satisfactory with fit indices of *χ*^2^ = 770.765 (*p* < 0.001), CFI = 0.944, SRMR = 0.040, and RMSEA = 0.076 (90% CI = 0.072–0.081). The ω of the PHQ-9 was 0.812, implying satisfactory internal consistency. The one-factor PHQ-9 had equivalent overall structure, factor loadings, and item intercepts across age groups, suggesting invariance across ages. Partial scalar invariance was demonstrated across sex and marital-status groups. Partial metric and scalar invariance were supported across education groups. Scalar invariance was supported among all of the medical-condition (hypertension, diabetes, cancer, arthritis, asthma, and heart disease) groups. Overall, the measurement invariance of the one-factor PHQ-9 was empirically supported across sociodemographic and medical-condition groups. The PHQ-9 can be reliably used to compare the severity of depressive symptoms across these groups in research and practice.

## Introduction

Depression is a common public health concern. It has been estimated that about 5% of adults suffer from depression worldwide, which impairs their daily functioning at work or in the family and adversely affects the quality of life, and may even result in suicide (World Health Organization, 2021). It also brings large economic costs related directly to the workplace (absenteeism and presenteeism) and to suicide (Greenberg et al., 2021). Early detection and prompt treatment are therefore important. For these reasons, the United States Preventive Services Task Force recommended screening for depression in the general adult population (Siu et al., 2016).

The Patient Health Questionnare-9 (PHQ-9) is a self-administered instrument that was developed to identify people who may have depression and assess the severity of depression symptoms in research and primary care (Kroenke et al., 2001). The PHQ-9 comprises nine items corresponding to the nine diagnostic criteria for depressive disorder in the fourth edition of the Diagnostic and Statistical Manual of Mental Disorders (DSM-IV) (American Psychiatric Association, 2000). When the PHQ-9 was initially developed using 6,000 patients across 8 primary-care and 7 obstetrics-gynecology clinics, it demonstrated satisfactory internal consistency, test–retest reliability, and convergent validity. Using a score criterion of ≥10 for major depression produced a sensitivity of 88% and specificity of 88%. The PHQ-9 was subsequently psychometrically examined in diverse populations (e.g., people with chronic disease, the elderly, college students, and adults) and languages (El-Den et al., 2018; Carroll et al., 2020). The PHQ-9 has been considered one of the most widely used self-reported measures in various primary-care settings worldwide (El-Den et al., 2018). The PHQ-9 has also been used in nationally representative health surveys, such as the National Health and Nutrition Examination Survey (NHANES) in the United States (Centers for Disease Control and Prevention, 2020), the Peruvian Demographic and Health Survey (Villarreal-Zegarra et al., 2019), the UK Biobank (Davis et al., 2020), and the Korea National Health and Nutrition Examination Survey (KNHANES) in South Korea (Korea Centers for Disease Control and Prevention, 2022).

Despite the widespread use of the PHQ-9, its psychometric property of measurement invariance has received little attention (Teymoori et al., 2020). Measurement invariance refers to the psychometric equivalence of a construct across groups and demonstrates that the construct has the same meaning among groups (Polit and Yang, 2016). Evidence for measurement invariance across the groups is needed when comparing differences in PHQ-9 scores between groups in research and practice, since a lack of such evidence could mean that the group difference is due to other measurement aspects rather than representing a true difference in depressive symptoms (Patel et al., 2019). This may result in the under- or over detection of people with depression in certain groups.

The measurement invariance of the PHQ-9 has been evaluated across sociodemographic groups (e.g., sex, age, marital status, education level, employment status, and race/ethnicity groups) in the United States, Spain, Germany, Bangladesh, and Portugal (Petersen et al., 2015; González-Blanch et al., 2018; Patel et al., 2019; Villarreal-Zegarra et al., 2019; Lamela et al., 2020; Rahman et al., 2022). The measurement invariance of the PHQ-9 has rarely been evaluated across medical-condition groups, so it remains unclear whether the meanings of the PHQ-9 items are similar between groups with and without a specific medical condition (e.g., disease). Nevertheless, differences in the PHQ-9 among medical-condition groups have been continuously reported. For example, depressive symptoms measured using the PHQ-9 were compared between patients with cancer and a general population (Hinz et al., 2016), between normotensive and prehypertensive groups (Jang, 2021), and between nonstroke and stroke survivor groups (Hong et al., 2021) without the evidence of measurement invariance in the PHQ-9 across these groups. In other words, the findings of group differences in depressive symptoms were insufficient for the confidence that the differences were due to the true nature of depression. Two recent studies evaluated the measurement invariance of the PHQ-9 across people with and without diabetes (Nouwen et al., 2021) and adults with and without HIV (Mwangi et al., 2020). More validation is needed to determine the measurement invariance of the PHQ-9 across groups with various medical conditions.

The aim of this study was therefore to determine the measurement invariance of the PHQ-9 across various sociodemographic (age, sex, marital status, and education level) and medical condition (hypertension, diabetes, cancer, arthritis, asthma, and heart disease) groups using a nationally representative Korean database. According to the COnsensus-based Standards for the selection of health Measurement Instruments (COSMIN), evidence of structural validity and internal consistency in a self-reported instrument are prerequisites when examining measurement invariance (Prinsen et al., 2018). Thus, the structural validity and internal consistency of the PHQ-9 were also assessed as the prerequisite properties for measurement invariance.

## Methods

### Study design and participants

A secondary data analysis study was conducted to psychometrically evaluate the PHQ-9 using a dataset from the KNHANES VIII-2, which was a cross-sectional nationwide survey conducted by the Korea Centers for Disease Control and Prevention (KCDC) (Korea Centers for Disease Control and Prevention, 2022). The KNHANES VIII-2 used a stratified multistage cluster sampling method to obtain a sample representative of the population. The protocol used for the survey comprised three components: (a) physical and laboratory examinations by a health professional in a mobile examination car, (b) a health survey administered using interviews and self-reported questionnaires in a mobile examination car, and (c) a nutritional survey using interviews in home visits. The first-two components were used in the present study. The KNHANES VIII-2 investigated 7,359 people from 3,314 households in 180 survey districts, 7,096 of whom participated in the first two components (Korea Centers for Disease Control and Prevention, 2022). Of them, 5,347 people who were older than 19 years and had completed at least 80% of the PHQ-9 items were finally included in the present study.

### Ethical considerations

Data collection for the KNHANES VIII-2 was permitted by an institutional review board of the KCDC (approval no. 2018-01-03-2C-A). The data were publicly released in 2022. This study was exempted from requiring informed consents by the Institutional Review Board at Ajou University Hospital (approval no. AJOUIRB-EX-2022-397).

### Measures

#### PHQ-9

The PHQ-9 (Kroenke et al., 2001) is a self-administered instrument that was developed to screen or assess the severity of depressive symptoms in primary-care settings. Each item is scored on a 4-point Likert scale with response options from 0 (“not at all”) to 3 (“nearly every day”) that refer to events during the previous 2 weeks. Total scores range from 0 to 27, with higher scores indicating more-severe depressive symptoms. The PHQ-9 demonstrated satisfactory internal consistency (Cronbach’s alpha = 0.89) and good sensitivity and specificity in identifying cases of major depressive disorders. The PHQ-9 was administered to obtain data for the KNHANES VIII-2 using face-to-face interviews. Sample weighting was not applied in the present study because the aim was to determine the measurement invariance of the PHQ-9.

#### Sociodemographic variables

Data on age, sex, marital status, and education level were collected from the self-reported health survey data set of the KNHANES VIII-2. Age was grouped into <65 and ≥ 65 years; sex into male and female; marital status into living with a spouse, divorced/widowed/separated, and never married/single; and education level into graduated from elementary school, middle school, high school, and college or above.

#### Medical condition variables

In the physical laboratory examinations of the KNHANES VIII-2, hypertension was defined as a systolic blood pressure of ≥140 mmHg, a diastolic blood pressure of ≥90 mmHg, taking a medication for high blood pressure. Prehypertension was defined as a systolic blood pressure of ≥120 and < 140 mmHg or a diastolic blood pressure of ≥80 and < 90 mmHg. Normal was defined as a systolic blood pressure of <120 mmHg and a diastolic blood pressure of <80 mmHg. Diabetes was diagnosed as a fasting blood glucose level of ≥126 mg/L, receiving a hypoglycemic agent/insulin injection, being diagnosed by a doctor, or having HbA1c ≥6.5%. Prediabetes was defined as a fasting blood glucose level of ≥100 and ≤ 125 mg/L or HbA1c ≥5.7 and < 6.4%. Normal was defined as a fasting blood glucose level of <100 mg/L or HbA1c <5.7%. Other medical-condition variables (cancer, arthritis, asthma, and heart disease) were classified into groups with and without the disease, based on the self-reported physician diagnosis in the health survey data set of the KNHANES VIII-2.

### Statistical analysis

Data were analyzed using SPSS for Windows (version 25), AMOS software (version 25), and the *EGAnet* package in the R environment. For the cross-validation approach to the structural validity of the PHQ-9, the total data were split into two subsamples using the random assignment function of SPSS. Subsample 1 (*n* = 2,673) was used for exploratory graph analysis (EGA) using the *EGAnet* package to investigate the underlying dimensionality of the PHQ-9. EGA is a recently developed method for investigating the number of dimensions (Golino and Epskamp, 2017). EGA was applied in the present study using the graphical least absolute shrinkage and selection operator (LASSO) method with the Walktrap community detection algorithm. This process graphed a network model, and edge weights were calculated. The graphical model was visually presented using nodes (items) and edges (links) that indicated correlations between two nodes after controlling for all other nodes in the network. EGA detects the number of dimensions (communities) by arranging densely clustered nodes into each dimension. The nodes are colored according to their identified communities. The network model is visually depicted with the weight matrices represented by the edges between the nodes.

The detected dimensionality of the PHQ-9 was further assessed through a nonparametric bootstrap procedure with 1,000 iterations using the *bootEGA* function. This analysis calculated the structural consistency (the proportion of times that each dimension derived from EGA is exactly recovered from the replicate bootstrap samples) and item stability (the proportion of times that a given item belongs to the same dimension obtained in the EGA in the bootstrap replications) (Golino and Christensen, 2022). Network loadings, which refers the association of each node with the dimension in which it belongs, were then obtained by using the *net.loads* function and interpreted as small (0.00–0.15), moderate (0.16–0.25), or large (0.26–0.35) loadings (Christensen and Golino, 2021a).

Confirmatory factor analysis (CFA) was subsequently conducted using AMOS to test the fit of the structure identified in the PHQ-9 network by the EGA in subsample 2 (*n* = 2,674). The CFA model was estimated using 1,000 bootstrap samples due to the assumption of the multivariate normality not being satisfied based on a Mardia’s coefficient estimate of >5.00 (Byrne, 2016). The goodness of fit of the model was assessed using multiple indices: comparative fit index (CFI), standardized root-mean-square residual (SRMR), and root-mean-square error of approximation (RMSEA). CFI values greater than 0.95 indicate a good fit, and values of 0.90–0.95 are considered to indicate an acceptable fit (Hu and Bentler, 1999). RMSEA and SRMR values less than 0.05 indicate a good fit, and those of 0.05–0.08 indicate an adequate fit (MacCallum et al., 1996). The traditional χ^2^ value and the number of degrees of freedom were also reported, but they were not used to determine the model fit since they are sensitive to the sample size (Schreiber, 2008).

With the total sample, the internal consistency of the PHQ-9 was assessed using omega coefficient ( ω ) with a criterion value of >0.70 (McDonald, 1999). Measurement invariance of the PHQ-9 across various sociodemographic and medical-condition groups was tested using multigroup CFA (MGCFA). There are four levels of invariance tests that progress in a hierarchical bottom-up approach: (a) configural invariance, when the number of latent constructs and the specific items loaded on them are assumed to be equivalent across groups, (b) metric invariance, when factor loadings from items to factors are assumed to be equal across groups, (c) scalar invariance, when factor loadings and item intercepts are assumed to be equal across groups, and (d) error variance invariance, when the error terms of items are assumed to be equal across groups in addition to the equality of the scalar invariance. The error variance invariance is considered to be excessively stringent and is often not achieved in practice (Chen and Tang, 2006), and so the first three levels of invariance tests were successively conducted in this study. In each progression, the higher model level was accepted if the value from the CFA decreased by <0.010, supplemented by changes in RMSEA (∆RMSEA) of <0.015 and SRMR (∆SRMR) of <0.030 (for metric invariance) or < 0.150 (for scalar invariance) (Chen, 2007). If a full metric or scalar invariance was not met, partial invariance was tested using the process of freeing factor loadings or item intercepts to detect noninvariant items.

## Results

### Descriptive statistics of study variables

The characteristics of the 5,347 participants are listed in Table 1. They comprised 54.8% (*n* = 2,931) females, and 15.2% (*n* = 1,349) were aged 65 years and older (age for the total sample = 51.26 ± 17.04 years). Participants living with their spouse comprised 65.2% (*n* = 3,486), and those with college education or above comprised 28.3% (*n* = 2,050). Participants diagnosed with hypertension and diabetes comprised 31.9% (*n* = 1,705) and 15.1% (*n* = 809), respectively. Participants who reported that they had been diagnosed with cancer, arthritis, asthma, and heart disease by a physician comprised 5.7, 12.9, 3.4, and 3.2%, respectively.

**Table 1:** Characteristics of the study participants.

Variable	Groups	Total sample (*n* = 5,347)	Subsample 1 (*n* = 2,673)	Subsample 2 (*n* = 2,674)
		*n* (%)	*n* (%)	*n* (%)
Sex	Male	2,416 (45.2)	1,198 (44.8)	1,218 (45.4)
	Female	2,931 (54.8)	1,475 (55.2)	1,456 (54.5)
Age	<65 years	3,998 (74.8)	1989 (74.4)	2009 (75.1)
	≥65 years	1,349 (25.2)	684 (25.6)	665 (24.9)
Marital status	Living with a spouse	3,486 (65.2)	1721 (64.4)	1765 (66.0)
	Divorced/widowed/separated	741 (13.9)	385 (14.4)	356 (13.3)
	Never married/single	1,117 (20.9)	567 (21.2)	550 (20.6)
Education level	Graduated from elementary school	899 (16.8)	448 (16.8)	451 (16.9)
	Graduated from middle school	517 (9.7)	249 (9.3)	268 (10.0)
	Graduated from high school	1880 (35.2)	940 (35.2)	940 (35.2)
	Graduated from college or above	2050 (28.3)	1,036 (38.8)	1,014 (37.9)
Hypertension	Normal	2,177 (40.7)	1,087 (40.7)	1,090 (40.8)
	Prehypertension	1,406 (26.3)	700 (26.2)	706 (26.4)
	Hypertension	1705 (31.9)	855 (32.0)	850 (31.8)
Diabetes	Normal	2,221 (41.5)	1,100 (41.2)	1,121 (41.9)
	Prediabetes	2,101 (39.3)	1,055 (39.5)	1,046 (39.1)
	Diabetes	809 (15.1)	408 (15.3)	401 (15.0)
Cancer^a^	No	5,043 (94.3)	2,509 (93.9)	2,534 (94.8)
	Yes	304 (5.7)	164 (6.1)	140 (5.2)
Arthritis	No	4,659 (87.1)	2,322 (86.9)	2,337 (87.4)
	Yes	688 (12.9)	351 (13.1)	337 (12.6)
Asthma	No	5,164 (98.0)	2,585 (96.7)	2,579 (96.4)
	Yes	183 (3.4)	88 (3.3)	95 (3.6)
Heart disease^b^	No	5,175 (96.8)	2,587 (96.8)	2,588 (96.8)
	Yes	172 (3.2)	86 (3.2)	86 (3.2)

a Stomach, liver, colon, breast, cervix, lung, thyroid, and other cancers.

b Myocardial infarction and angina.

### Prerequisites for measurement invariance: structural validity

#### Dimensionality by EGA with subsample 1

**Figure 1:**
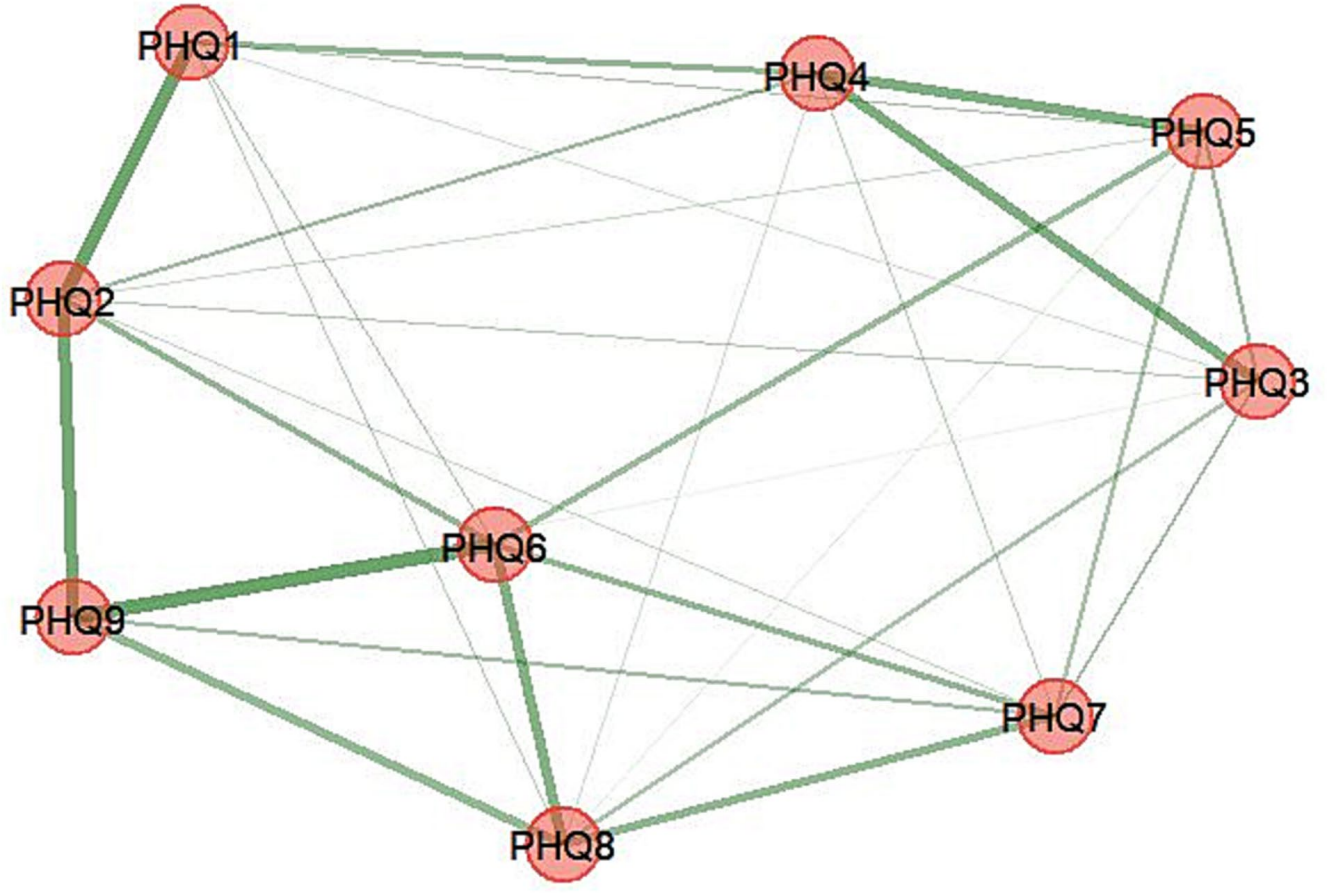
Exploratory graph analysis: the dimensional structure of the PHQ-9. Item 1: anhedonia; item 2: depressed mood; item 3: sleep disturbance; item 4: fatigue; item 5: appetite changes; item 6: low self-esteem; item 7: concentration difficulties; item 8: psychomotor disturbances; item 9: suicide ideation.

The EGA detected one dimension (communality) of nodes that are depicted using identical colors in Figure 1, suggesting that one dimension contained all nine items. The edge weights (partial correlations between nodes) are presented in Supplementary Table S1. The highest edge weight was between items 6 and 9; in other words, these item pairs exhibited relatively stronger associations. The dimensionality structure of the PHQ-9 using *bootEGA* indicated that one dimension (median network structure = 1) was identified in 100% of the bootstrap iterations. That is, the structural consistency of the one-dimensional solution for the PHQ-9 was stable because the replication of >75% or more bootstrap samples is considered to exhibit adequate structural consistency (Golino et al., 2021).

If an item stability value is less than 0.80 (80%), it may be problematic (Christensen and Golino, 2021b). All of the item stability values in this study were > 0.80 in the bootstrap replications (Supplementary Figure S1), and no unstable item needed to be removed from the PHQ-9. The network loading values were between 0.398 and 0.256 (Supplementary Table S2), which were interpreted as large loadings (>0.025) on their dimension.

#### Dimensionality by CFA with subsample 2

**Figure 2:**
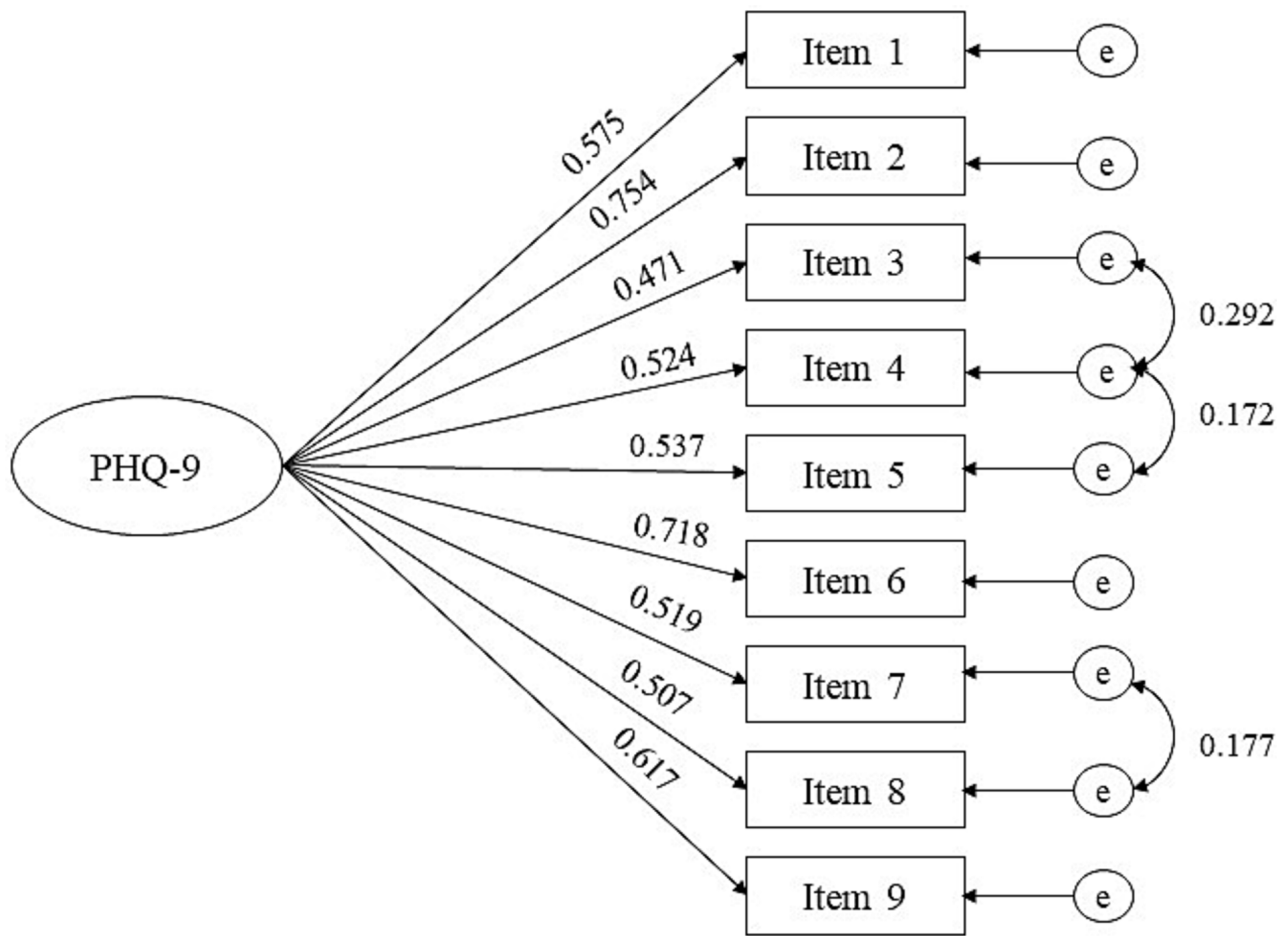
Confirmatory factor analysis model of the PHQ-9 with subsample 2. Item 1: anhedonia; item 2: depressed mood; item 3: sleep disturbance; item 4: fatigue; item 5: appetite changes; item 6: low self-esteem; item 7: concentration difficulties; item 8: psychomotor disturbances; item 9: suicide ideation; *e*, measurement error.

CFA with subsample 2 was conducted to verify the one-dimension structure of the PHQ-9 identified by EGA with subsample 1. The initial CFA mode of the PHQ-9 partially met the fit indices: χ^2^ = 897.529 (*p* < 0.001), CFI = 0.878, SRMR = 0.059, and RMSEA = 0.110 (90% confidence interval [CI] = 0.140–0.116). The model was therefore modified, and the model fit was substantially improved (∆CFI > 0.10) (Byrne, 2016) when the three pairs of error terms were allowed to be correlated: *χ*^2^ = 398.205 (*p* < 0.001), CFI = 0.948, SRMR = 0.040, and RMSEA = 0.076 (90% CI = 0.070–0.083) (Table 2). Factor loading values are presented in Figure 2.

**Table 2:** Summary of fit indices in CFA of the PHQ-9.

Model	χ^2^	*df*	CFI	SRMR	RMSEA (90% CI)	ΔCFI
Initial model	897.529*	27	0.878	0.059	0.110 (0.104–0.116)	
Modified model 1^a^	606.745*	26	0.919	0.049	0.091 (0.085–0.098)	0.041
Modified model 2^b^	479.763*	25	0.936	0.045	0.082 (0.076–0.089)	0.017
Modified model 3^c^	398.205*	24	0.948	0.040	0.076 (0.070–0.083)	0.012

df, degrees of freedom; CFI, comparative fit index; SRMR, standardized root-mean-square residual; RMSEA, root-mean-square error of approximation; Δ CFI, change in CFI compared with the previous model; CI, confidence interval.

a Covariance between measurement errors of items 3 and 4.

b Covariance between measurement errors of items 3 and 4, and items 7 and 8.

c Covariance between measurement errors of items 3 and 4, items 7 and 8, and items 4 and 5.

*p < 0.01.

### Prerequisites for measurement invariance: internal consistency

The omega coefficients of the PHQ-9 with subsample 1, subsample 2, and the total sample were 0.803, 0.821, and 0.812, respectively, implying satisfactory internal consistency.

### Measurement invariance

The single structural model of the PHQ-9 that was validated by EGA and CFA was also satisfactory in a total sample, with fit indices of *χ*^2^ = 770.765 (*p* < 0.001), CFI = 0.944, SRMR = 0.040, and RMSEA = 0.076 (90% CI = 0.072–0.081). The measurement invariance of the structural PHQ-9 model across each sociodemographic and medical-condition group is presented in Table 3. Regarding age, configural invariance was supported by the model fit indices (CFI = 0.939, RMSEA = 0.058, and SRMR = 0.046) across age groups (<65 vs. ≥65 years). Metric and scalar invariance also met the criteria for ∆CFA, ∆RMSEA, and ∆SRMR. These results demonstrated that the PHQ-9 had consistent overall structure, factor loadings, and item intercepts across age groups. Associated with sex, scalar invariance was not supported (∆CFA = 0.012). To detect invariant item intercepts, partial scalar invariance was assessed. As a result, the partial scalar invariance model with the freely estimated item-3 intercept was supported across sex groups. There was also scalar noninvariance (∆CFA = 0.016) in the PHQ-9 across three marital-status groups (living with a spouse vs. divorced/widowed/separated vs. never married/single). By freeing the intercepts of items 3 and 1, the partial scalar invariance model was supported across the marital groups. The matric invariance of the PHQ-9 was not supported in education level (∆CFA = 0.014). The factor loadings of item 9 were not equal across the education-level groups. Partial metric invariance was supported by freeing the factor loadings of item 9. If partial metric invariance is not achieved, a serious measurement problem occurs and the next level of the invariance test cannot proceed (Collier, 2020). Since the partial metric invariance was achieved across education-level groups in the present study, the next level of the scalar invariance test was performed and supported. Regarding the medical conditions (hypertension, diabetes, cancer, arthritis, asthma, and heart disease), it was found that the configural, metric, and scalar invariance of the PHQ-9 were all supported for the groups with and without each medical condition (all ∆CFA < 0.010). As an ancillary analysis, the measurement invariance of the PHQ-9 was tested across two groups: one group without disease (*n* = 2,675) and one group with at least one of hypertension, diabetes, cancer, arthritis, asthma, or heart disease (*n* = 2,519). The results supported the presence of configural, metric, and scalar invariance (all Δ CFI < 0.010).

**Table 3:** Measurement invariance of the PHQ-9 across sociodemographic and medical-condition groups.

Invariance model	χ^2^	*df*	CFI	RMSEA	SRMR	ΔCFI	ΔRMSEA	ΔSRMR
**Age**								
Configural	899.084*	48	0.939	0.058	0.046			
Metric	958.360*	56	0.934	0.055	0.047	0.005	0.004	0.001
Scalar	1062.353*	65	0.927	0.054	0.047	0.007	0.001	0.001
**Sex**								
Configural	863.040*	48	0.940	0.056	0.042			
Metric	927.944*	56	0.935	0.054	0.045	0.005	0.002	0.003
Scalar	1103.657*	65	0.923	0.055	0.045	0.012	0.001	< 0.001
Partial scalar^a^	1045.595	64	0.927	0.054	0.045	0.008	< 0.001	< 0.001
**Marital status**								
Configural	851.518*	72	0.938	0.045	0.039			
Metric	944.781*	88	0.932	0.043	0.043	0.006	0.002	0.004
Scalar	1158.035*	106	0.916	0.043	0.043	0.016	< 0.001	< 0.001
Partial scalar^b^	1072.313	102	0.923	0.042	0.043	0.009	0.001	< 0.001
**Education level**								
Configural	1114.890*	96	0.926	0.045	0.036			
Metric	1332.682*	120	0.912	0.043	0.060	0.014	0.002	0.024
Partial metric^c^	1250.648*	117	0.918	0.045	0.051	0.008	< 0.001	0.015
Partial scalar^c^	1401.356*	144	0.909	0.051	0.061	0.009	0.006	0.010
**Hypertension**								
Configural	880.393*	72	0.940	0.046	0.046			
Metric	1031.226*	88	0.930	0.045	0.056	0.010	0.001	0.010
Scalar	1158.268*	106	0.922	0.043	0.056	0.008	0.002	< 0.001
**Diabetes**								
Configural	896.021*	72	0.935	0.047	0.045			
Metric	958.169*	88	0.932	0.044	0.049	0.003	0.003	0.004
Scalar	1008.220*	106	0.929	0.041	0.049	0.003	0.003	< 0.001
**Cancer**								
Configural	919.936*	48	0.936	0.058	0.041			
Metric	954.613*	56	0.934	0.055	0.041	0.002	0.003	< 0.001
Scalar	967.608*	65	0.933	0.051	0.041	0.001	0.004	< 0.001
**Arthritis**								
Configural	813.453*	48	0.943	0.055	0.039			
Metric	899.793*	56	0.937	0.053	0.040	0.006	0.001	0.001
Scalar	969.593*	65	0.932	0.051	0.041	0.003	< 0.001	0.001
**Asthma**								
Configural	838.504*	48	0.940	0.056	0.041			
Metric	865.377*	56	0.938	0.052	0.041	0.002	0.004	0.002
Scalar	896.186*	65	0.937	0.049	0.041	0.004	0.001	< 0.001
**Heart disease**								
Configural	849.517*	48	0.940	0.056	0.039			
Metric	915.561*	56	0.936	0.054	0.040	0.004	0.002	0.001
Scalar	930.792*	65	0.936	0.050	0.040	< 0.001	0.004	< 0.001
**Nonexistence and existence of disease** ^d^								
Configural	795.436*	48	0.942	0.055	0.045			
Metric	887.944*	56	0.935	0.053	0.052	0.007	0.002	0.007
Scalar	985.494*	65	0.929	0.052	0.052	0.006	0.001	<0.001

df, degrees of freedom; CFI, comparative fit index; SRMR, standardized root mean square residual; RMSEA, root mean square error of approximation; Δ, change in the value compared with that in the previous model.

*p < 0.001.

a Item intercept for item 3 was not constrained.

b Item intercepts for items 3 and 1 were not constrained.

c Item loadings for item 9 were not constrained.

d At least one of hypertension, diabetes, cancer, arthritis, asthma, or heart disease.

## Discussion

### Prerequisites for measurement invariance: structural validity and internal consistency

When developing a self-reported scale, the most basic step is the conceptualization of the construct to be measured, and the underlying structure of that construct is assessed based on the defined conceptualization (Polit and Yang, 2016). The PHQ-9 was originally developed by turning the diagnosis criteria of the DSM-IV into self-reported items without not only conceptualization but also structural validity, even though its internal consistency, test–retest reliability, and convergent validity were satisfactory (Kroenke et al., 2001). This revealed that the factorial structure of the PHQ-9 was inconsistent. According to a systematic review, 19 of 33 studies (57.6%) examined the structural validity using a CFA-supported 1-factor structure, and 12 studies (36.4%) supported a 2-factor structure (with different types of item clustering) (Lamela et al., 2020). According to psychometric studies (Arrieta et al., 2017; Alpizar et al., 2018; Keum et al., 2018; Boothroyd et al., 2019), both one- and two-dimension models provided good fits in CFA. However, a one-dimension structure was finally chosen as providing appropriate structural validity of the PHQ-9 because the correlation between factors in the two-dimension structure was high ( φ >0.80). Stochl et al. (2022) stated that the structural inconsistency is mainly due to different sample properties and methodologies. The few studies that have examined the structural validity of the PHQ-9 in South Korean populations also demonstrated one- or two-factor structures. Two studies demonstrated a one-factor structure using exploratory factor analysis (EFA) among patients with heart failure or gastrointestinal symptoms (Lee et al., 2014) and CFA among university students (Kim and Lee, 2019). Other two studies found two-factor structures using EFA (Park, 2017) or using both EFA and CFA (Shin et al., 2020) in general populations, and the patterns of clustering items into factors differed among them.

In general, CFA, which confirms a hypothesized factor structure by using a theory or empirical evidence, is known to provide more-compelling evidence for structural validity than EFA (Polit and Yang, 2016). When the construct to be measured has no theoretical rationale of dimensions, EFA is used to provide empirical evidence for the CFA measurement model. In this case, both EFA and CFA are used for structural validity, which is called a cross-validation approach. EGA has recently emerged as a powerful assessment tool for identifying the number of factors that underlie multivariate data in network psychometrics (Golino and Epskamp, 2017). In a simulation study, EGA demonstrated greater accuracy than traditional factor analysis methods (e.g., EFA) in estimating the number of latent factors (Golino et al., 2020). EGA was therefore used instead of EFA to assess the number of factor structures in the PHQ-9 in the present study, which demonstrated a one-factor structure. The one-factor structure has also been demonstrated using EGA in patients living with epilepsy (Sebera et al., 2020).

In this study, the empirically derived one-factor structure of the PHQ-9 using EGA had satisfactory structural validity in the CFA. The one-factor structure in the present study has been previously demonstrated among nationally representative general populations in Peru (*n* = 30,449) (Villarreal-Zegarra et al., 2019) and European countries (Ui, *n* = 2,025; Ireland, *n* = 1,041; Spain, *n* = 1,949; and Italy, *n* = 1,048) (Shevlin et al., 2022). Some researchers insisted that the number of factor structures was likely to be one when the PHQ-9 was applied to a more-heterogeneous sample (e.g., a general population) because the item variance would be greater and thus items would load on one factor (Petersen et al., 2015). However, the PHQ-9 was demonstrated to have a two-factor structure in data representative of the United States population from the 2005–2016 NHANES (Patel et al., 2019). The inconsistency in the number of factors therefore might not only be explained by the hetero- or homogeneous characteristics of a sample; the era and culture of the society and environment in which people are currently living may also be considered as the possible source of this inconsistency. For example, the content of item 6 (“Trouble concentrating on things, such as reading the newspaper or watching television”) might not have been a problem when the PHQ-9 was developed in 2001 (Kroenke et al., 2001). The internet penetration rate is 97% (DataReportal, 2021) and the rate of smartphone use among adults is 95% (Pew Research Center, 2019) in South Korea. The rapid adoption of internet and device technologies has resulted in the daily newspaper utilization rate decreasing from 87.8% in 1993 to 44.6% in 2011 and 8.9% in 2021, and over-the-top (OTT) media services were introduced in 2013 in South Korea (Korea Press Foundation, 2021). In other words, most South Koreans do not read the daily newspapers, and the pattern of watching TV has been moving from terrestrial television broadcasting toward OTT media services that can be watched anytime, anywhere, and with any device. In these conditions, the content of item 6 may lead to biased responses. It is therefore recommended to adjust the phrasing of the item 6 (“…reading the newspaper or watching television”) to correspond to the current circumstances.

The omega coefficient indicated that the internal consistency of the PHQ-9 was satisfactory in this study. Cronbach’s alpha has been dominantly used to assess the internal consistency of a self-reported questionnaire. However, this metric criticized due to the violation of tau-equivalence, and the omega coefficient has emerged as a new alternative (Taylor, 2021). The omega value of the PHQ-9 was also supported by studies involving a general population in Peru (ω = 0.87) (Villarreal-Zegarra et al., 2019) and university students in Bangladesh (ω = 0.86) (Rahman et al., 2022).

### Measurement invariance across sociodemographic and medical-condition groups

The measurement invariance of the PHQ-9 across age groups was supported in the present study. This was congruent with studies involving general populations in other countries (Villarreal-Zegarra et al., 2019; Lamela et al., 2020). The consistency of this finding implies that the depressive symptoms scored by the PHQ-9 can be meaningfully compared among age groups in a general population.

Invariance has been reported in the PHQ-9 across sexes in the general population of the United States (Patel et al., 2019), and across primary-care patients in Spain (González-Blanch et al., 2018) and in Germany (Petersen et al., 2015). In the present study, partial scalar invariance across sex was yielded by the noninvariant intercept of item 3 (“Trouble falling, staying asleep or sleeping too much”). However, the effect of the partial scalar invariance on comparing mean differences between groups is small and is not practically relevant (Schuler et al., 2018). Considering this, the PHQ-9 was able to yield invariance across sex groups with minimal risk of bias in the present study. Partial scalar invariance was also yielded across marital groups. This finding was consistent with those of other studies involving general populations in Portugal and Peru (Villarreal-Zegarra et al., 2019; Lamela et al., 2020).

The PHQ-9 has been demonstrated to be invariant across education-level groups in a general population and in primary-care patients (González-Blanch et al., 2018; Patel et al., 2019; Lamela et al., 2020). However, the PHQ-9 in the present study yielded a partial metric invariant model across four education-level groups after freeing (unconstraining) the factor loading of item 9 (“Thought that you would be better off dead, or of hurting yourself in some way”). That is, the meaning of the item 9 differed across the education-level groups. South Korea is well known as a country in which educational competition is very higher and educational achievement is very important (Kwak and Ickovics, 2010). Because people with lower education levels are more likely to experience financial constraints, and being poor is known to be a major reason for suicide in the country (Kim et al., 2010; Lee et al., 2017; Hong et al., 2021), the suicide rate has been ranked the highest among the Organization for Economic Cooperation and Development (OECD) countries (OECD, 2022). People with different education levels therefore seem to respond differently to item 9 about suicide ideation.

The noninvariance of item 9 in this study might have also occurred due to the potential for response bias. While other studies administered the PHQ-9 using an internet or paper-pencil mode (González-Blanch et al., 2018; Patel et al., 2019; Lamela et al., 2020), the PHQ-9 survey was performed in this study using face-to face interviews. Since item 9 is a very sensitive question, respondents might not answer it frankly in an interview. Another potential reason is the content of the item 9 itself. Controversy exists regarding the item because its content of self-harm is not part of the nine criteria for depressive symptoms from the DSM that had used in the development of the PHQ-9 (Kroenke et al., 2009; Wu et al., 2020). For this reason, the PHQ-8 omits item 9 from the PHQ-9 and can be utilized in a large general population, such as in the Behavioral Risk Factor Surveillance System survey in the United States (Kroenke et al., 2009) It is therefore recommended to use self-reported paper-and-pencil/internet modes rather than an interview mode for the PHQ-9, or to consider using the PHQ-8 with item 9 omitted if the PHQ-8 has satisfactory psychometric properties in a specific population.

The meaning of the PHQ-9 in the present study was equivalent for people with and without each medical condition (hypertension, diabetes, cancer, arthritis, asthma, and heart disease), which suggests that researchers and health professionals can use the PHQ-9 to reliably compare between groups. The measurement invariance across medical-condition groups has rarely been studied. It is therefore further recommended to test such psychometric validation in various disease groups.

### Limitations

The data analyzed in this study were collected using a cross-sectional design, and so the measurement invariance of the PHQ-9 could not be examined over time. It is therefore recommended to assess whether the one-factor model of the PHQ-9 is invariant over time. In contrast to the hypertension and diabetes groups, people with and without cancer, arthritis, asthma, and heart disease were determined using self-reported physical diagnoses that might induce bias in the diagnostic accuracy. The samples of the medical-condition groups (asthma and heart disease) were too small, and so their findings of invariance should be interpreted with caution. This was the secondary analysis of a large data set, so the other psychometric examinations (e.g., test–retest reliability, convergent validity, criterion validity, and responsiveness) were not available for the PHQ-9.

## Conclusion

The one-factor model of the PHQ-9 confirmed in this study empirically supported its measurement invariance across various sociodemographic and medical-condition groups. In other words, the meaning of the PHQ-9 was similar to people across the groups. Therefore, the PHQ-9 can be reliably used to compare the severity of depressive symptoms across the groups in research and practice.

## Data availability statement

Publicly available datasets were analyzed in this study. This data can be found here: data used in this study are available on the website Korea National Health & Nutrition Examination Survey at https://knhanes.kdca.go.kr/knhanes/sub03/sub03_02_05.do.

## Ethics statement

The studies involving humans were approved by Institutional Review Board at Ajou University Hospital. The studies were conducted in accordance with the local legislation and institutional requirements. Written informed consent for participation was not required from the participants or the participants' legal guardians/next of kin in accordance with the national legislation and institutional requirements.

## Author contributions

E-HL: study conception and design, data analysis and interpretation, draft preparation, manuscript writing and editing, and approval of the submitted version of the manuscript. EK, and H-JK: data screening, literature review and manuscript editing, and approval of the submitted version of the manuscript. HL: data analysis and interpretation, manuscript editing, and approval of the submitted version of the manuscript. All authors contributed to the article and approved the submitted version.

## Conflict of interest

The authors declare that the research was conducted in the absence of any commercial or financial relationships that could be construed as a potential conflict of interest.

## Publisher’s note

All claims expressed in this article are solely those of the authors and do not necessarily represent those of their affiliated organizations, or those of the publisher, the editors and the reviewers. Any product that may be evaluated in this article, or claim that may be made by its manufacturer, is not guaranteed or endorsed by the publisher.
