# Supplementary material for: Measurement invariance of the patient health questionnaire-9 depression scale in a nationally representative population-based sample
Source: Front Psychol. 2023 Aug 31;14:1217038. doi: 10.3389/fpsyg.2023.1217038 (PMC10500305; doi:10.3389/fpsyg.2023.1217038)
Supplement: Supplementary file 1 [file Data_Sheet_1.docx]

**Supplementary Material**

**Supplementary Table 1** Network edge weights estimated using the LASSO

| Items | Item 1 | Item 2 | Item 3 | Item 4 | Item 5 | Item 6 | Item 7 | Item 8 | Item 9 |
| --- | --- | --- | --- | --- | --- | --- | --- | --- | --- |
| Item 1 | – |  |  |  |  |  |  |  |  |
| Item 2 | 0.32 | – |  |  |  |  |  |  |  |
| Item 3 | 0.24 | 0.07 | – |  |  |  |  |  |  |
| Item 4 | 0.17 | 0.13 | 0.24 | – |  |  |  |  |  |
| Item 5 | 0.09 | 0.04 | 0.13 | 0.24 | – |  |  |  |  |
| Item 6 | 0.08 | 0.16 | 0.00 | 0.00 | 0.14 | – |  |  |  |
| Item 7 | 0.00 | 0.04 | 0.13 | 0.06 | 0.10 | 0.17 | – |  |  |
| Item 8 | 0.06 | 0.00 | 0.10 | 0.03 | 0.01 | 0.23 | 0.19 | – |  |
| Item 9 | 0.00 | 0.29 | 0.01 | 0.00 | 0.00 | 0.33 | 0.12 | 0.18 | – |

Item 1: Anhedonia; Item 2: Depressed mood; Item 3: Sleep disturbance; Item 4: Fatigue; Item 5: Appetite changes; Item 6: Low self-esteem; Item 7: Concentration difficulties; Item 8: Psychomotor disturbances; Item 9: Suicide ideation.

**Supplementary Table 2** EGA dimensionality and network loadings of the PHQ-9

| Items | Dimension 1 |
| --- | --- |
| Item 6 | 0.398 |
| Item 2 | 0.380 |
| Item 9 | 0.338 |
| Item 4 | 0.313 |
| Item 8 | 0.292 |
| Item 7 | 0.288 |
| Item 5 | 0.277 |
| Item 1 | 0.270 |
| Item 3 | 0.256 |

Item 1: Anhedonia; Item 2: Depressed mood; Item 3: Sleep disturbance; Item 4: Fatigue; Item 5: Appetite changes; Item 6: Low self-esteem; Item 7: Concentration difficulties; Item 8: Psychomotor disturbances; Item 9: Suicide ideation.


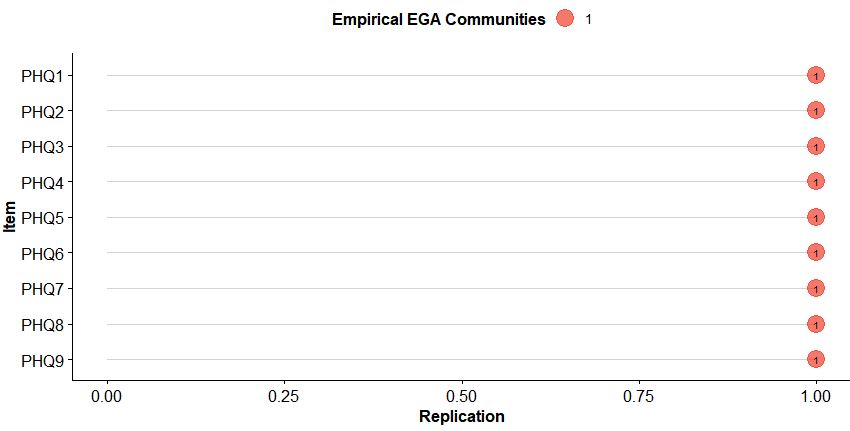


**Supplementary Figure 1** Item stability in the one-dimensional structure of the PHQ-9.

Item 1 (PHQ1): Anhedonia; Item 2 (PHQ2): Depressed mood; Item 3 (PHQ3): Sleep disturbance; Item 4 (PHQ4): Fatigue; Item 5 (PHQ5): Appetite changes; Item 6 (PHQ6): Low self-esteem; Item 7 (PHQ7): Concentration difficulties; Item 8 (PHQ8): Psychomotor disturbances; Item 9 (PHQ9): Suicide ideation.
